# Brain-Computer Interface Controlled Functional Electrical Stimulation System for Ankle Movement

**DOI:** 10.1186/1743-0003-8-49

**Published:** 2011-08-26

**Authors:** An H Do, Po T Wang, Christine E King, Ahmad Abiri, Zoran Nenadic

**Affiliations:** 1 Department of Neurology, University of California, Irvine, CA 92697 USA; 2 Department of Neurology, Long Beach Veterans Affairs Medical Center, Long Beach, CA 90822 USA; 3 Department of Biomedical Engineering, University of California, Irvine, CA 92697 USA; 4 Department of Electrical Engineering and Computer Science, University of California, Irvine, CA 92697 USA

## Abstract

**Background:**

Many neurological conditions, such as stroke, spinal cord injury, and traumatic brain injury, can cause chronic gait function impairment due to foot-drop. Current physiotherapy techniques provide only a limited degree of motor function recovery in these individuals, and therefore novel therapies are needed. Brain-computer interface (BCI) is a relatively novel technology with a potential to restore, substitute, or augment lost motor behaviors in patients with neurological injuries. Here, we describe the first successful integration of a noninvasive electroencephalogram (EEG)-based BCI with a noninvasive functional electrical stimulation (FES) system that enables the direct brain control of foot dorsiflexion in able-bodied individuals.

**Methods:**

A noninvasive EEG-based BCI system was integrated with a noninvasive FES system for foot dorsiflexion. Subjects underwent computer-cued epochs of repetitive foot dorsiflexion and idling while their EEG signals were recorded and stored for offline analysis. The analysis generated a prediction model that allowed EEG data to be analyzed and classified in real time during online BCI operation. The real-time online performance of the integrated BCI-FES system was tested in a group of five able-bodied subjects who used repetitive foot dorsiflexion to elicit BCI-FES mediated dorsiflexion of the contralateral foot.

**Results:**

Five able-bodied subjects performed 10 alternations of idling and repetitive foot dorsifiexion to trigger BCI-FES mediated dorsifiexion of the contralateral foot. The epochs of BCI-FES mediated foot dorsifiexion were highly correlated with the epochs of voluntary foot dorsifiexion (correlation coefficient ranged between 0.59 and 0.77) with latencies ranging from 1.4 sec to 3.1 sec. In addition, all subjects achieved a 100% BCI-FES response (no omissions), and one subject had a single false alarm.

**Conclusions:**

This study suggests that the integration of a noninvasive BCI with a lower-extremity FES system is feasible. With additional modifications, the proposed BCI-FES system may offer a novel and effective therapy in the neuro-rehabilitation of individuals with lower extremity paralysis due to neurological injuries.

## Background

Many neurological conditions, such as stroke, spinal cord injury (SCI), and traumatic brain injury (TBI), can leave the affected individual with severe or complete paralysis. There are currently no biomedical treatments available that can reverse the loss of motor function after these neurological injuries [1], and physiotherapy typically provides only a limited degree of motor function recovery [2-4]. Brain-computer interface (BCI) is a relatively novel technology with the potential to restore, substitute, or augment lost motor behaviors in patients with devastating neurological conditions such as high-cervical SCI or amyotrophic lateral sclerosis [5-8]. For example, BCIs systems have enabled direct brain control of applications such as computer cursors [8], virtual keyboards [9,10], and movement within virtual reality environments [11-13]. Most notably, BCIs have enabled the direct brain control of limb prosthetic devices [7,14], and such BCI-controlled prostheses represent a promising neuro-rehabilitative technology for motor function restoration in the neurologically injured. In the future, they may provide a permanent solution for restoration of lost motor functions, especially if no equivalent biomedical treatment exists.

Generally, BCI control of a limb prosthesis is accomplished by acquiring neurophysiological signals associated with a motor process, analyzing these signals in real time, and subsequently translating them into commands for a limb prosthesis. To date, this concept has been successfully applied to the control of robotic arms [15] and functional electrical stimulation (FES) devices of the upper extremities [7,14]. More specifically, Hochberg et al. [15] demonstrated how a subject with tetraplegia due to SCI could use an invasive BCI to operate a robotic arm to perform a simple task of moving an object from one point to another and to open and close a robotic hand. Also, Pfurtscheller's group [7,14] demonstrated how an individual affected by tetraplegia due to SCI was able to utilize a noninvasive electroencephalogram (EEG)-based BCI to control hand grasping via FES to complete a goal-oriented task of grasping an object and moving it another location.

In spite of encouraging results achieved with upper extremity BCI-FES systems, the integration of BCI with lower extremity FES systems has received less attention. At the time of this publication, review of the literature revealed that no actual BCI-FES systems for the lower extremities have been reported on. This may be partly explained through historical reasons, as BCI system development has been primarily focused on individuals with severe paralysis, such as those with locked-in syndrome or high cervical SCI [16]. These individuals would most likely benefit from using BCI technology that restores communication and upper extremity function for interaction with the environment. Meanwhile, wheeled mobility has generally been considered an effective and robust method of substitution for ambulation in lower extremity paralysis. Finally, in the context of EEG-based BCIs, lower extremity movements, such as ambulation, may cause significant artifacts which in turn may require the use of specialized EEG systems (e.g. active or actively shielded electrodes), thus creating a research barrier for laboratories without this technology.

Focusing the development of BCI technology on individuals with complete paralysis due to neurological injury significantly limits its application domain. Recently, BCI-FES systems are increasingly being explored as potential neuro-rehabilitation tools for improving partially impaired upper extremity function in individuals with stroke [17], thereby vastly broadening the potential target population. Given that an estimated 36% of stroke patients [4], 68% of SCI patients [18,19], and 61% of TBI patients [20] are affected by significant chronic gait impairment, there is a compelling need for the development of BCI-FES system for the lower extremities. Furthermore, the development of such a system may facilitate neural plasticity and repair mechanisms to improve impaired lower extremity and gait functions in these patient populations. This will not only further broaden the application domain of BCI technology, but will also yield a novel neuro-rehabilitation approach to some of the most prevalent neurological injuries. As the initial step towards achieving this goal, we describe the first integration of a noninvasive EEG-based BCI with a noninvasive FES system that enables the direct brain control of foot dorsifiexion. The performance of the system was tested in a small group of able-bodied subjects who were able to use repetitive foot dorsifiexion to elicit BCI-FES mediated dorsifiexion of the contralateral foot.

## Methods

### Overview

**Figure 1:**
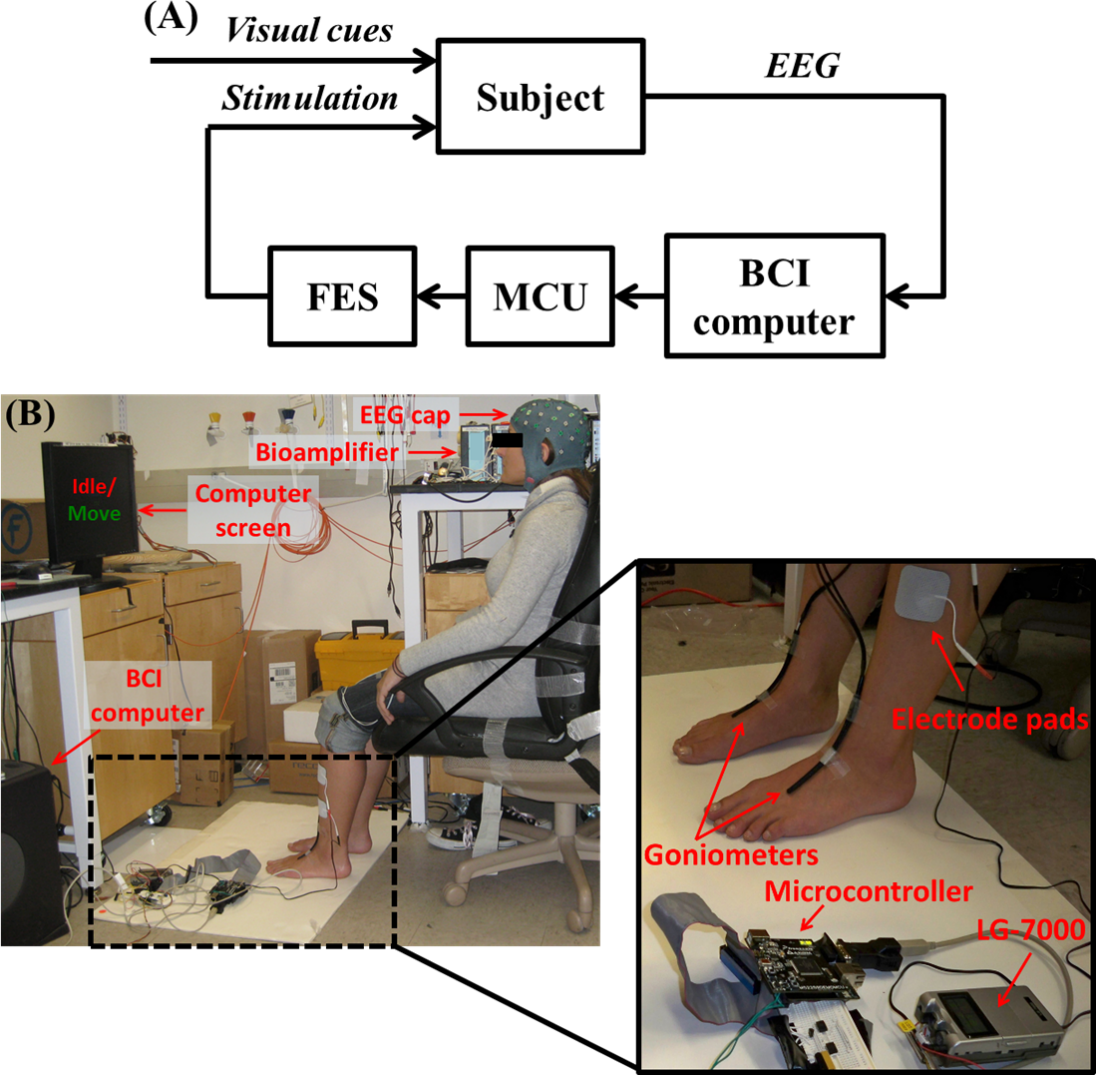
**Integrated BCI-FES system**. (A) Block diagram of the integrated BCI-FES system. In response to visual cues, the subject performs actions (idling or dorsifiexion), the underlying EEG data are analyzed by a BCI computer, and instructions are sent to a microcontroller unit (MCU). The MCU controls an FES system that sends feedback to the subject by means of stimulation. (B) Experimental setup showing the subject performing right foot dorsifiexion in response to visual cues displayed on the computer screen. EEG signals underlying this activity are recorded by the EEG cap and sent to the bioamplifier, and then to the BCI computer for analysis. The computer sends commands to a commercial Food & Drug Administration (FDA) approved FES device by means of the MCU. The FES device then stimulates the TA muscle of the foot, thereby causing contralateral dorsifiexion. The inset shows the MCU connected to the neuromuscular stimulator and the placement of surface FES electrodes. Also visible is a pair of custom-made electrogoniometers [22], used for measurement of both executed and BCI-FES mediated foot dorsifiexion.

The goal of this study is to integrate a noninvasive EEG-based BCI system with a noninvasive FES system for the lower extremities. The schematic diagram of the overall system is shown in Figure 1A. The proposed system utilizes a contralaterally-controlled FES paradigm [21], wherein healthy subjects perform repetitive foot dorsifiexion, EEG patterns underlying this action are detected in real time, and this information is subsequently used to trigger FES of the tibialis anterior (TA) muscle of the contralateral foot so as to achieve its dorsifiexion. The study entails a training procedure, where preliminary EEG data is collected and a subject-specific prediction model is designed, followed by an online session, where the real-time performance of the integrated BCI-FES system is tested.

### Recruitment

The study was approved by the Institutional Review Board of the University of California, Irvine. Since the present work represents a proof-of-principle study, it was aimed at able-bodied subjects who are generally healthy with no history of neurological conditions. Five subjects were recruited and provided their informed consent to participate in the study. Their demographic data are shown in Table 1.

**Table 1:** Population Demographics

Subject	Sex	Age (yr)	Dominant Side	BCI Experience (hr)
1	F	24	L	20
2	M	40	R	10
3	M	29	R	5
4	M	28	R	0
5	F	56	R	5

The demographics of five able-bodied subjects. The columns list: subject number, sex, age, dominant side (L-left, R-right), and number of hours of relevant BCI experience.

### Signal Acquisition

An actively-shielded EEG cap (MediFactory BV, Heerlen, the Netherlands) with 64 sintered Ag-AgCl electrodes, arranged according to the 10-20 International Standard, was used for EEG recording (see Figure 1B). Conductive gel (Compumedics USA, Charlotte, NC) was applied to all electrodes and the 30-Hz impedances between each electrode and the reference electrode were maintained at <10 Ω by abrading the scalp with a blunt needle. The EEG signals were amplified, band-pass filtered (0.01-50 Hz), digitized (sampling rate: 256 Hz, resolution: 22 bits), and acquired in a common average reference mode using two linked 32-channel bioamplifiers (NeXus-32, Mind Media, Roermond-Herten, the Netherlands). A pair of custom-made electrogoniometers [22] were mounted onto the anterior surface of each ankle and were used to measure foot dorsifiexion (see Figure 1B). The goniometer traces were acquired by a data acquisition system (MP150, Biopac Systems, Goleta, CA) with a sampling rate of 4 kHz and a resolution of 16 bits. Both the data acquisition and experimental protocols were controlled by custom-made Matlab (Mathworks, Natick, MA) scripts. EEG data recorded during training procedures were saved for offline analysis, while those recorded during online sessions were analyzed in real time (see below).

### Training Procedure

To achieve BCI control of the FES device and in turn control foot dorsifiexion, the BCI system must be able to reliably decode EEG signals associated with either foot dorsifiexion or idling. To this end, a prediction model was synthesized by first recording EEG signals during alternating epochs of foot dorsifiexion and idling. More specifically, each subject was seated in a chair, approximately 0.8 to 1 m from a computer monitor, which displayed instructional cues during all experimental procedures (see Figure 1B). Subjects were then instructed to alternate between 6-sec epochs of idling and repeated foot dorsifiexion. The frequency of dorsifiexion was determined by the subject and ranged between 6 and 9 dorsifiexion cycles per 6-sec epoch (1.0-1.5 Hz). A total of 200 epochs (100 epochs per class) were performed, with the procedure lasting approximately 20 min. Finally, the above training procedure was repeated using the opposite foot and the foot that yielded the prediction model with the highest classification accuracy (see Offline Signal Analysis and Prediction Model Generation section below) was chosen to continue with the remainder of the study.

### Offline Signal Analysis and Prediction Model Generation

Channels whose EEG signals were excessively contaminated by electromyogram (EMG) artifacts were excluded from analysis. To this end, an iterative artifact rejection algorithm was used, where channels whose EEG amplitude exceeded an outlier voltage threshold in more than 25% of the total trials were removed. The outlier threshold was nominally set to 6 standard deviations (SD) from the mean, and was adaptively changed to keep the number of outlier trials below a pre-specified number (5% of all trials in the present study). The above procedure was repeated until no more channels could be removed. To minimize the effect of outliers on statistical estimates, robust (i.e. median-based) mean and standard deviation were used [23]. The above procedure typically resulted in the exclusion of signals from circumferential "hat band" electrodes which usually overlay the mastoid process, the forehead, the occiput, and the temporalis muscles. Upon artifact removal, a continuous 20-min EEG record was split into 100 idle and 100 dorsifiexion trials based on the corresponding electrogoniometer signals recorded simultaneously with EEG during the training procedure. Each EEG trial (~6 sec) was then transformed into the frequency domain using the Fast Fourier Transform (FFT), and its power spectral density was integrated in 2 Hz bins centered at 1, 3, 5, · · ·, 49 Hz. This resulted in 25 binned power spectral values per channel. A frequency search was then performed to find the best contiguous frequency range for classification. Initially, the full range of frequencies (0.01-50 Hz) was used, resulting in a 25 × *C *dimensional data matrix, where *C *is the number of retained EEG channels (*C *ranged between 44 and 46 across all subjects). To facilitate subsequent classification, the dimension of input data was reduced using a combination of classwise principal component analysis (CPCA) [24,25] and approximate information discriminant analysis (AIDA) [26]. This resulted in the extraction of one-dimensional (1D) spatio-spectral features:

(1) $$f=T_{A}\Phi_{C}\left(d\right)$$

where **d **∈ ℝ^25×*C *^is single-trial EEG data, **Φ**_C _: ℝ^25*×C *^→ ℝ*^m ^*is a piecewise linear mapping from the data space into an *m*-dimensional CPCA-subspace, and **T**_A _: ℝ*^m ^*→ ℝ is an AIDA transformation matrix. A detailed description of CPCA, AIDA, and a related information-theoretic feature extraction technique can be found in [25-27], respectively. A linear Bayesian classifier:

(2) $$\frac{P\left(I|f^{⋆}\right)}{P\left(D|f^{⋆}\right)}\begin{array}{c} I\\ >\\ <\\ D \end{array}1$$

was then designed in the feature domain, where $P\left(I|f^{⋆}\right)$ and $P\left(D|f^{⋆}\right)$ are the posterior probabilities^1 ^of idling and dorsifiexion classes, respectively. Equation (2) is read as: "classify *f*^⋆ ^as idling class if $P\left(I|f^{⋆}\right)>P\left(D|f^{⋆}\right)$, and vice versa." The performance of the Bayesian classifier (2), expressed as classification accuracy, was then assessed by performing 5 runs of a stratified 10-fold cross-validation [28].

The lower bound of the frequency range was then increased in 2-Hz steps, and the above procedure was repeated until the classifier performance stopped improving. This defined the optimal lower frequency bound, *F_L_*. Once *F_L _*was found, the optimal higher frequency bound, *F_H_*, was found in a similar manner. The parameters of the prediction model, including the optimal frequency range, the feature extraction mapping, and the classifier parameters, were then saved for real-time EEG analysis necessary for online BCI-FES operation. Finally, the signal processing, feature extraction, and classification algorithms were implemented into the BCI software for real-time operation.

### Online Signal Analysis

During online operation, 0.5 sec segments of EEG data were acquired in real time at a frequency of two non-overlapping segments per second. The EEG data segments were then processed as described in the previous section. Briefly, the EEG signals were band-pass filtered and the data from the artifact prone channels were removed. The remaining data were transformed into the frequency domain by FFT, and the power spectral densities (over the optimal frequency range) were calculated. The spectral data were then used as an input for the feature extraction algorithm, which resulted in the extraction of 1D spatio-spectral features. The posterior probabilities of idling and dorsifiexion classes given the observed EEG features, were then calculated as described in the previous section.

### BCI-FES Integration

**Figure 2:**
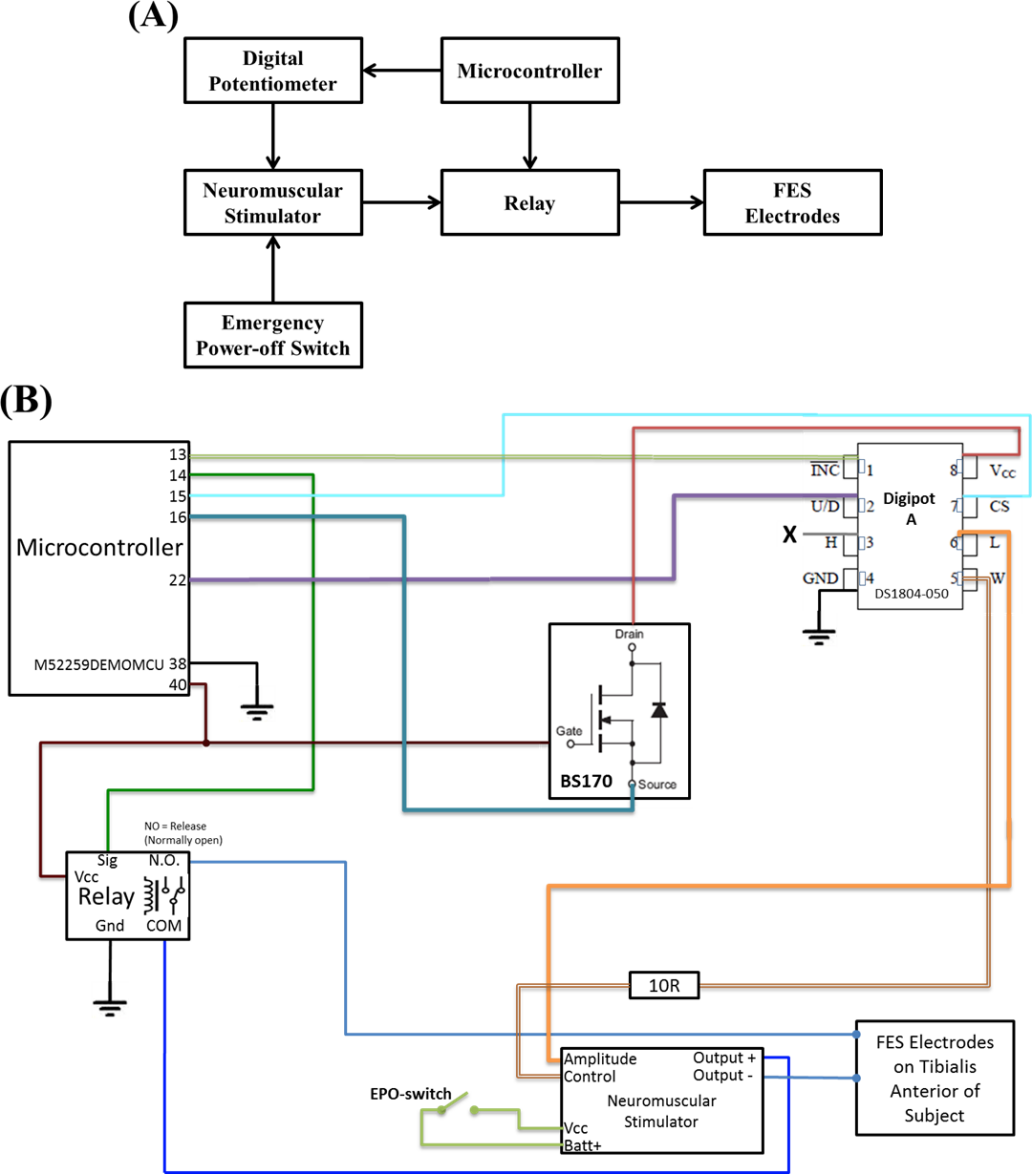
**BCI-FES control module**. (A) The block diagram shows a microcontroller unit (MCU) interfaced with a digital potentiometer (digipot) and a relay. The digipot modulates the amplitude of the stimulating current, while the relay keeps the circuit between the surface FES electrodes and the stimulator normally open. The relay circuit closes when it receives a logical high from the MCU (coinciding with the detection of dorsifiexion state by the BCI computer). For safety reasons, a manually operated emergency power-off (EPO) switch is added to the stimulator power supply circuit. (B) The circuit diagram of the BCI-FES control module showing detailed wiring scheme. The digipot's resistance changes from 0 kΩ to 50 kΩ, thereby changing the amplitude of the stimulating current from 0 mA to 100 mA. Not shown in (A) is a field-effect transistor (BS170), used to ensure proper power-on sequence for the digipot.

A low-cost, FDA-approved, constant-current neuromuscular stimulator (LG-7000, LG Medical Supplies, Austin, TX) was used for functional electrical stimulation of the neuromuscular system consisting of the deep peroneal nerve and the TA muscle (see Figure 1B). To facilitate BCI-FES integration, the stimulator's manually controlled "on/off" switch and analog potentiometer that adjusted the amplitude of the stimulating current had to be modified to allow computer control of the stimulator (see Figure 2). To this end, the FES device's analog potentiometer was replaced with a digital potentiometer by utilizing a General Pin Input Output (GPIO) interface. Likewise, the switch function was emulated by using a digital relay that kept the stimulating circuit closed/open when electrical stimulation was/was not intended. Both the digital potentiometer and the relay were controlled by a microcontroller unit (Freescale M52259, Freescale Semiconductors, Austin, TX) in a master-slave configuration. More specifically, a custom-made C-language program was used to instruct the microcontroller unit (MCU) to listen for command requests from the BCI computer via a DB9 serial port, utilizing a universal asynchronous receiver/transmitter protocol. These requests carried the information on whether to turn the stimulator "on" or "off" (as determined by the prediction model), and the intensity of electrical stimulation (as determined by the experimenter). Based on the current relay and potentiometer states, the MCU generated the appropriate signals needed to achieve the desired result. For example, when real-time EEG data were classified as "dorsifiexion," the BCI software sent a series of instructions to the MCU that commanded the relay to close the stimulation circuit and the digital potentiometer to decrease its resistance, thereby initiating electrical stimulation. This continued until the real-time EEG data were decoded as "idle," upon which the BCI software sent a series of instructions to the MCU to open the relay, thereby opening the stimulation circuit and stopping the electrical stimulation. During operation, the BCI-FES system toggled between these two states.

### Calibration

**Figure 3:**
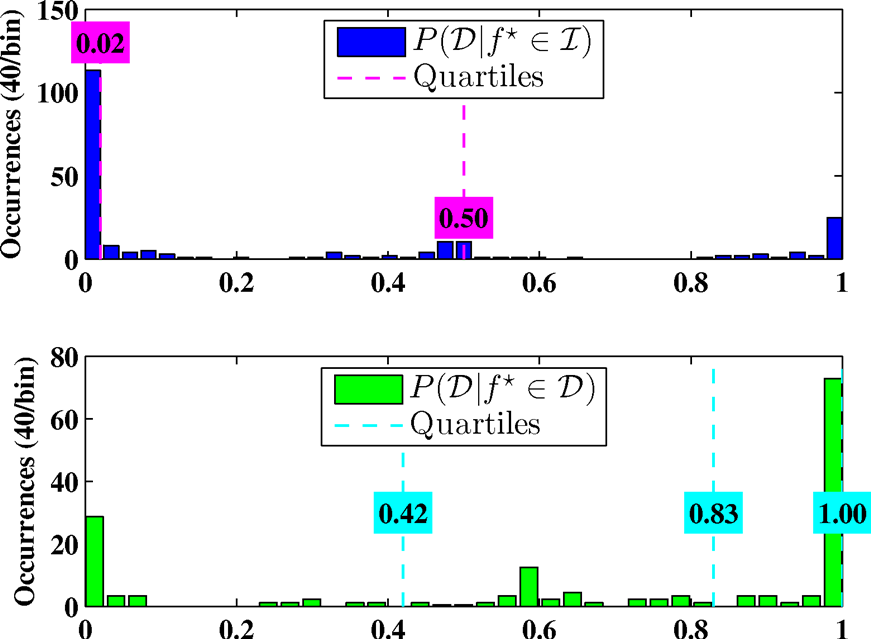
**Histograms of the posterior class probabilities for subject B**. Based on the known underlying action (idling or dorsifiexion), the distributions of the posterior probabilities, $P\left(D|f^{⋆}\in I\right)$ and $P\left(D|f^{⋆}\in D\right)$, are empirically estimated as histograms. Dashed lines indicate the 25%, 50%, and 75% quartiles, where the 25% and 50% quartiles for $P\left(D|f^{⋆}\in I\right)$ overlap. Note that $P\left(D|f^{⋆}\in I\right)=1-P\left(I|f^{⋆}\in I\right)$.

**Figure 4:**
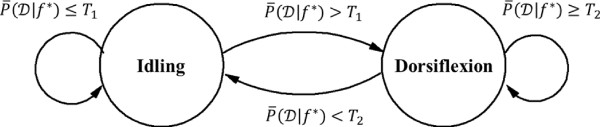
**Finite state machine diagram of the online BCI-FES system operation**. The BCI-FES system is a binary state machine with idling and dorsifiexion states represented by circles. The state transitions are represented by the arrows, with transitions triggered by the conditions shown next to the arrows. The transitions are executed every 0.5 sec. Self-pointing arrows denote that the system remains in the present state.

Prior to online BCI operation, a brief calibration procedure was performed to determine the posterior probability thresholds for optimal online BCI-FES operation so that the number of false state transitions is minimized. Using the prediction model based on the training data, the BCI-FES system was set to run in the online mode without FES stimulation. Subjects were prompted to alternate between 20-sec epochs of idling and repetitive foot dorsifiexion for a total of 3 min. Meanwhile, real-time EEG signal analysis was performed, and the posterior probabilities of dorsifiexion and idling given data, $P\left(D|f^{⋆}\right)$ and $P\left(I|f^{⋆}\right)$, were calculated every 0.5 sec, as described in Online Signal Analysis section. The distributions of the posterior probabilities, $P\left(D|f^{⋆}\in I\right)$ and $P\left(D|f^{⋆}\in D\right)$, were then empirically estimated as in Figure 3. Since the BCI-FES system is a binary state machine, two thresholds were chosen from the histograms--one to trigger the transitions from "idle" to "dorsifiexion" state $\left(T_{1}=median\;P\left(D|f^{⋆}\in D\right)\right)$, and another for the transitions from "dorsifiexion" to "idle" state $\left(T_{2}=median\;P\left(D|f^{⋆}\in I\right)\right)$. During online BCI operation, the posterior probabilities $P\left(D|f^{⋆}\right)$ were averaged over a 1.5 sec period, and the average probabilities $\bar{P}\left(D|f^{⋆}\right)$ were compared to the thresholds *T*_1 _and *T*_2_. Depending on the present state, the transitions of the BCI-FES system were governed by the rules as illustrated by the state-machine diagram in Figure 4.

### Online BCI-FES Evaluation

#### Experimental Procedure

To evaluate the performance of the BCI-FES dorsifiexion system, subjects engaged in a contralaterally-controlled FES paradigm, similar to that described in [21]. FES preparation included the application of self-adhesive surface electrodes to the skin over the anterior lateral lower leg, covering the approximate course of the deep peroneal nerve, as illustrated in Figure 1B. Test stimulation was used to confirm that the electrode placement and chosen stimulation parameters were adequate for effective foot dorsifiexion (*~*15° to 20°). The stimulation parameters, including current amplitude, pulse width, and frequency, were empirically determined to achieve the required foot dorsifiexion without causing discomfort to the subject.

To ascertain purposeful control of the BCI-FES system, subjects performed ten alternating 10-sec epochs of idling and repetitive dorsifiexion of the optimally chosen foot (see Training Procedure section) to induce BCI-FES mediated dorsifiexion of the contralateral foot. Since the present study focused on able-bodied subjects, an ipsilaterally controlled FES paradigm was not used due to the inability to resolve voluntary and BCI-FES mediated dorsifiexion. Instructions to perform this task were shown as textual cues on the computer screen. Both voluntary and BCI-FES mediated foot dorsifiexion were measured by electrogoniometers.

#### Performance Analysis

The analysis of online BCI-FES operation was performed by comparing the epochs of voluntary and BCI-FES mediated foot dorsifiexion. For this purpose, the readings from the two electrogoniometers (see Figure 1) were first smoothed by a 100-msec Gaussian window, and epochs of foot dorsifiexion and idling were determined by a threshold crossing. A time series, *x*, describing voluntary foot dorsifiexion was then defined as:

(3) $$x\left[i\right]=\left\{\begin{array}{cc} 0, & if\;i\in I\\ 1, & if\;i\in D \end{array}\right)$$

where *i *= 1, 2, · · ·, *N*, and *N *is the number of samples in the goniometer trace. A time series, *y*, describing BCI-FES mediated foot dorsifiexion, was defined in a similar manner. The normalized cross-covariance function between the time series *x *and *y *was then calculated as:

(4) $$\rho \left(m\right)=\frac{\sum_{i=1}^{N}\left(x\left[i+m\right]-\bar{x}\right)\left(y\left[i\right]-ȳ\right)}{\sqrt{\sum_{i=1}^{N}{\left(x\left[i\right]-\bar{x}\right)}^{2}}\sqrt{\sum_{i=1}^{N}{\left(y\left[i\right]-ȳ\right)}^{2}}}$$

where *m *∈ [-*N *+ 1, *N - *1] is the lag between the sequences *x *and *y*, and $\bar{x}$ and $\bar{y}$ are the sample means of the two sequences, respectively. The latency between voluntary and BCI-FES mediated foot dorsifiexion was then found as the lag with maximal cross-covariance, i.e. *m*^⋆ ^= arg max*_m _ρ*(*m*). Subsequently, the temporal correlation between *x *and *y *was found to be: *ρ*^⋆ ^= *ρ*(*m*^⋆^). In addition, the absence of a BCI-FES mediated foot dorsifiexion epoch initiated within the duration of any voluntary foot dorsifiexion epoch was considered an omission. Finally, the initiation of a BCI-FES mediated foot dorsifiexion epoch within any idling epoch was considered a false alarm.

## Results and Discussion

### Results

#### Offline Performance

Each subject underwent training data collection as described in the Methods section. The EEG data associated with epochs of idling and repetitive foot dorsifiexion were analyzed and classified using the prediction model generated from this analysis. The input data for the prediction model were the powers of multi-channel EEG signals calculated in 2-Hz bins. The optimal subject-specific EEG frequency bands (see Table 2) were found using the procedure described in the Methods section, and included the *μ *(8-13 Hz), *β *(13-30 Hz) and low-*γ *(30-38 Hz) bands for Subject 1, high-*β *(22-30 Hz) and low-*γ *(30-50 Hz) bands for Subject 2, *μ*, *β *and low-*γ *(30-50 Hz) bands for Subject 3, *μ *and *β *bands for Subject 4, and *μ*, *β *and low-*γ *(30-50 Hz) bands for Subject 5.

**Table 2:** Overall Performances

Subject	Foot	EEG-band (Hz)	Classification Accuracy	Current (mA)	Pulse Width (*μ*sec)	Frequency (Hz)	Lag (sec)	*ρ* ^⋆^	OM	FA
1	R	[8-38]	94.4%	100	140	20	3.1	0.67	0	0
2	L	[22-50]	97.6%	100	200	30	1.4	0.72	0	0
3	L	[8-50]	85.1%	90	200	30	2.7	0.59	0	1
4	R	[8-30]	91.9%	88	200	20	3.0	0.62	0	0
5	R	[10-50]	93.6%	100	120	20	2.9	0.77	0	0

The performances of five subjects. The columns list: the foot that was voluntarily dorsiflexed, the EEG frequency band that was used for classification, (offline) classification accuracy as established by 10-fold cross-validation, the stimulating current amplitude, its pulse width and frequency, (online) lag between voluntary and BCI-FES-mediated dorsifiexion epochs, temporal correlation between these epochs (*ρ*^⋆^) calculated at the corresponding lags, omissions (OM), and false alarms (FA).

The offline performance was evaluated by performing 10-fold cross-validation, and a classification accuracy ranging from 85.1% to 97.6% was achieved (see Table 2). These results are statistically significant, as the probability of achieving the performance ≥ 85%, i.e. correctly classifying 170 or more trials (out of 200) by random chance, is only 3.0866 × 10^-25^. Note that cross-validation provides a safeguard against prediction model overfitting by ensuring that classification accuracy observed offline generalizes to future online sessions.

**Figure 5:**
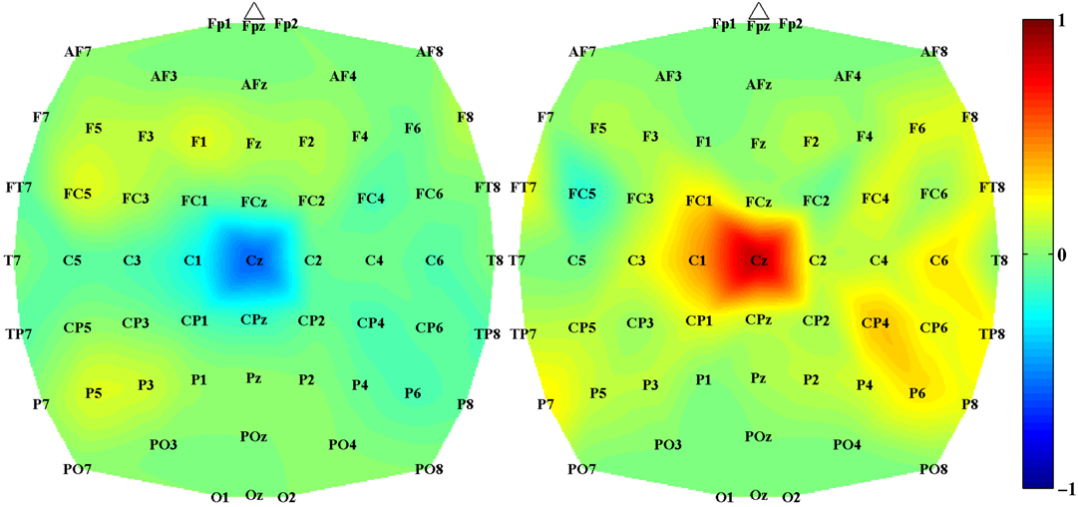
**Topographic distribution of spectral features**. Feature extraction mapping at high-*β *band (two-Hz bin centered at 29 Hz) for subject B. Values close to +1 and -1 indicate brain areas of importance for classifying EEG data into idling and dorsifiexion classes. Since our feature extraction mapping is piecewise linear, there are two maps; one adapted to idling class (left) and one adapted to dorsifiexion class (right). Note that both maps feature the area around the Cz-electrode as prominent, indicating the importance of this brain area at this particular frequency for distinguishing between idling and foot dorsifiexion.

**Figure 6:**
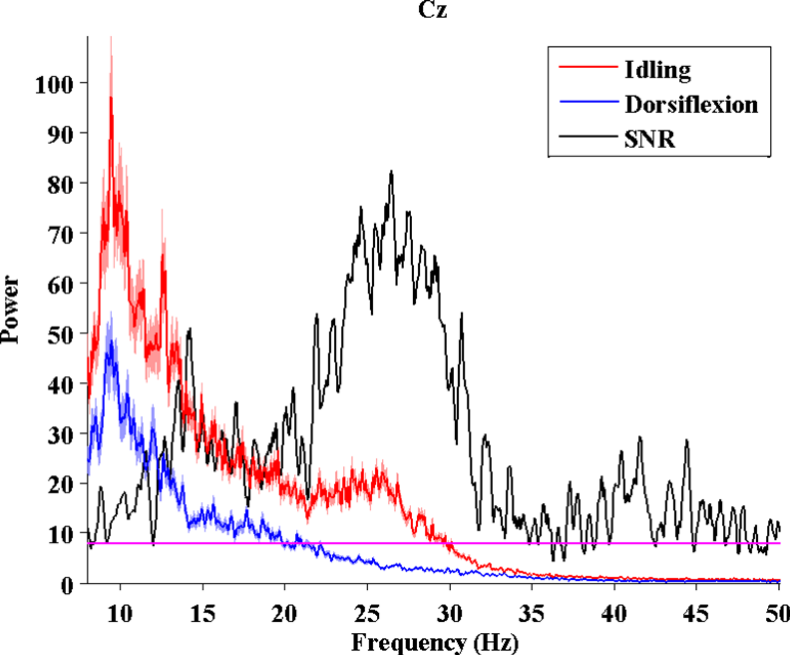
**Power spectral density at electrode Cz**. A broadband (8-50 Hz) desynchronization of EEG signals at electrode Cz for subject B. Red and blue traces denote the average (*n *= 100) power spectra of EEG signals under idling and foot dorsifiexion conditions, respectively. The shades represent ±1 SEM (standard error of mean) bounds. Black trace represents the signal-to-noise ratio (SNR), defined as in [36]: $\text{SNR(}f\text{)}\;\text{=}\frac{{\left(\mu_{\text{i}}(f)-\mu_{\text{d}}(f)\right)}^{2}}{\sigma_{\text{i}}^{2}(f)+\sigma_{\text{d}}^{2}(f)}$, where *f *is the frequency, *μ*_i_(*f*) and *μ*_d_(*f*) are the average powers at the frequency *f *under idling and dorsifiexion conditions, respectively, and $\sigma_{i}^{2}\left(f\right)$ and $\sigma_{d}^{2}\left(f\right)$ are the corresponding variances. The values of SNR above the magenta line define the frequencies with statistically significant difference between *μ*_i_(*f*) and *μ*_d_(*f*) (p *<*0.01, paired t-test).

Analysis of subject-specific prediction models demonstrated that the EEG power changes in the *β*-band observed over mid-central areas (i.e. electrode Cz) were the most informative features for classification (see Figure 5). These findings were confirmed by examining the power spectrum of EEG signals at Cz under both idling and dorsifiexion conditions (see Figure 6), where a prominent event-related desynchronization (loss of power) was observed over a broad frequency band. These observations are consistent with prior studies, where similar event-related desynchronization was observed upon initiation or imagination of movement [29-31].

#### Online BCI-FES Performance

Surface electrode placement for effective FES-induced dorsifiexion was confirmed prior to online BCI evaluation for all subjects. In general, stimulation parameters depend on skin impedance, muscle mass, and the subjects' electrical stimulation tolerance, and were therefore chosen empirically for each subject while ensuring that ~15°-20° of foot dorsifiexion was achieved. The subject-specific stimulation parameters are summarized in Table 2. In addition, prior to online BCI-FES evaluation, a test FES procedure was performed and no FES interference was visible on the EEG signals.

**Figure 7:**
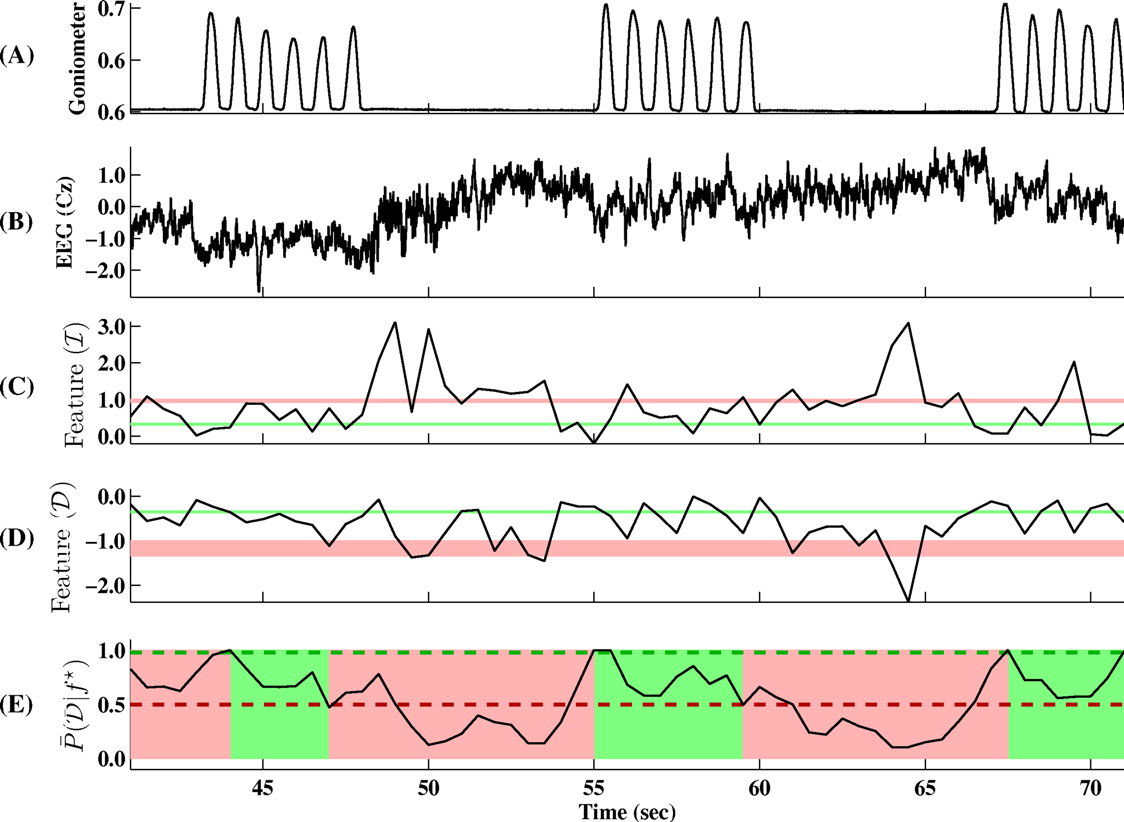
**Online EEG classification illustrated on training data**. (A) A goniometer trace delineating idling and dorsifiexion states. (B) The corresponding EEG signal trace recorded at the Cz electrode. (C),(D) One-dimensional spatio-spectral EEG features extracted using Eq. (1) shown in the subspaces corresponding idling (I) and dorsifiexion (D) states, respectively. The pink and green bands represent the mean ± 2 standard deviations (SD) of features corresponding to idling and dorsifiexion training data, respectively. (E) The average posterior probability of dorsifiexion given feature, *f*^⋆^. Dashed lines correspond to the thresholds, *T*_1 _(green) and *T*_2 _(red) as determined in the Calibration section. As outlined in Fig. 4, when the average posterior probability $\bar{P}\left(D|f^{⋆}\right)>T_{1}$, the BCI-FES system transitions to dorsifiexion state (shown as green block). Conversely, when $\bar{P}\left(D|f^{⋆}\right)<T_{2}$, the BCI-FES system transitions to idling state (pink block).

During online BCI-FES operation, each subject performed repetitive dorsifiexion of their optimally chosen foot to induce BCI-FES-mediated dorsifiexion of the contralateral foot. More specifically, each 0.5 sec segment of EEG data was acquired and analyzed as explained in the Methods section, and based on this analysis, the computer instructed the FES system to respond. The basic steps of this procedure applied to the training data are illustrated in Figure 7.

**Figure 8:**
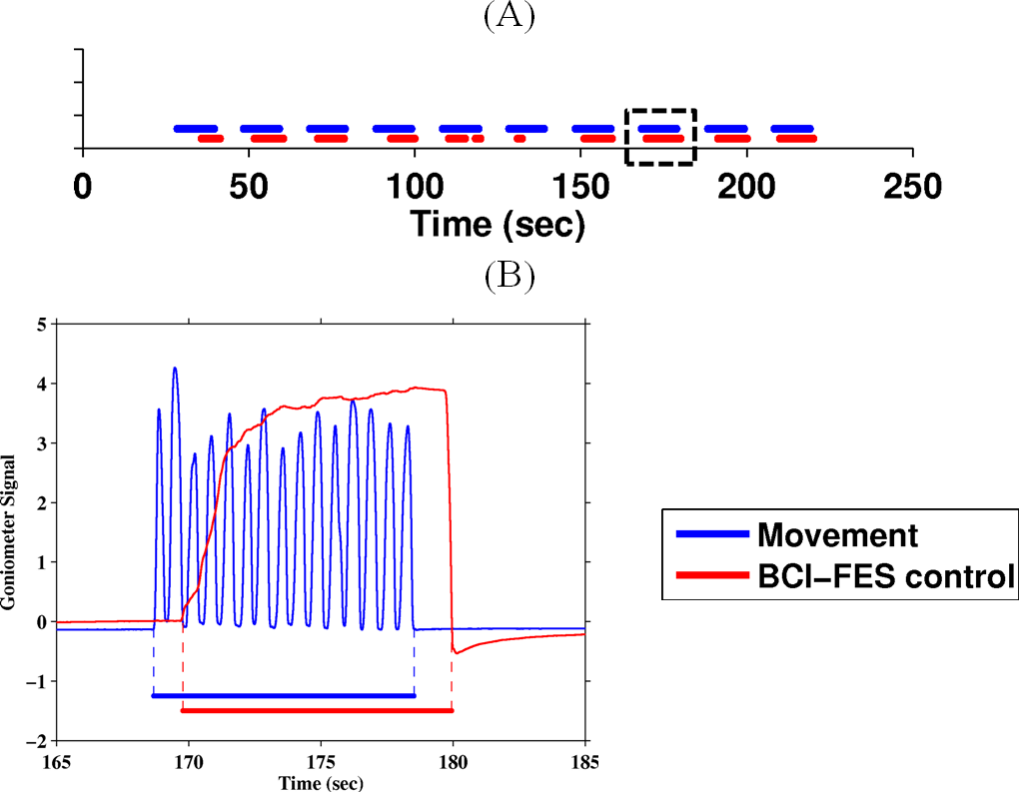
**Online performances of a representative subject**. (A) Blue trace marks the 10 epochs of 10-sec-long repetitive foot dorsifiexion for Subject 2, and red trace marks the epochs of BCI-FES mediated dorsifiexion of the contralateral foot. (B) The inset of a single dorsifiexion epoch [dashed box in (A)] showing the goniometer trace corresponding to 15 dorsifiexion cycles (blue) and BCI-FES-mediated dorsifiexion (red).

The online performances are quantified by four criteria: (i) lag between actual and BCI-FES-mediated dorsifiexion epochs, (ii) temporal correlation (at the corresponding lag value) between these epochs, (iii) number of omissions, and (iv) number of false alarms. Figure 8 shows the best online session for Subject 2.

All subjects performed the task with no omissions (100% BCI-FES response). However, BCI-FES-mediated dorsifiexion epochs typically lag behind the actual dorsifiexion epochs, and the average values of this latency ranged from 1.4 sec to 3.1 sec across all subjects (see Table 2). Temporal correlations between the voluntary and BCI-FES-mediated dorsifiexion epochs ranged between 0.59 and 0.77, and are also shown in Table 2. The statistical significance of these results was confirmed by running 10,000 Monte Carlo simulation trials with a chance level classification accuracy (50%). The maximum correlation coefficient obtained from the simulation was 0.41, and therefore even the lowest correlation coefficient of 0.59 is significant with a p-value *<*10^-4^.

The correlation coefficient measures the temporal consistency between voluntary foot dorsifiexion and the corresponding BCI-FES-mediated dorsifiexion response. Note that its value is normalized between -1 and 1, and appears to correlate with offline accuracy. For example, Subjects 2 and 5, who achieved the highest offline classification accuracy, also had the highest correlation coefficients. Conversely, Subject 3 achieved the lowest classiffication accuracy and correlation coefficient. This drop in online performance may be attributed to a single false alarm (see Table 2). Subjects 1, 2, 4 and 5, on the other hand, had no false alarms.

## Discussion

This study reports on the first successful integration of a noninvasive EEG-based BCI with a noninvasive FES system for the lower extremities. The performance of the integrated BCI-FES system was tested in a population of five able-bodied subjects, utilizing a contralaterally-controlled FES paradigm [21] where subjects performed repetitive dorsifiexion of their optimally chosen foot to trigger BCI-FES-mediated dorsifiexion of the contralateral foot. This paradigm was chosen since ipsilateral dorsifiexion and stimulation in able-bodied subjects would produce confounding results, as it would be difficult to resolve voluntary and BCI-FES-induced movements.

During the training procedures, the subjects were instructed to refrain from excessive face, mouth and eye movements. However, natural movements associated with normal seated behavior (eye blinks, swallowing, small eye and facial movements) were permitted. Note that these movements are not expected to cause any systematic error as long as they are not synchronized with either dorsifiexion or idling. To support this claim, Subject 4 was also fitted with electrooculogram (EOG) and EMG electrodes for simultaneous recording of eye and facial muscle movements during the training procedure. Analogous to EEG data, EMG/EOG data were used to design a prediction model. The performance of this classifier was 53%, which was not statistically different (p-value: 0.22) from the chance level performance (50%). In summary, since idling and dorsifiexion could not be predicted from EMG/EOG signals, it is thus extremely unlikely that EEG was contaminated by EOG/EMG artifacts in a systematic manner. Finally, the active shielding feature of our EEG system minimized the electromagnetic interference due to cable movements and mechanical vibrations.

Offline analysis of EEG signals corresponding to epochs of repetitive foot dorsifiexion and idling collected during the training procedures revealed that the EEG power in the *μ*, *β *and low-*γ *bands were responsible for encoding the differences between idling and dorsifiexion states. The change in the signal power was mostly observed over the mid-central area, which likely corresponds to activity within the primary motor cortex's foot representation area (located in the interhemispheric fissure of the brain) and/or supplementary motor area. This was further confirmed by examining the feature extraction maps of the prediction models (see Figure 5), which indicated that mid-central brain areas played a prominent role in classifying idling and dorsifiexion states. While these results are not surprising from a brain anatomy standpoint, it should be noted that our prediction model is entirely data driven, and so these observations underscore the physiological and anatomical plausibility of our feature extraction map. It should also be noted that these spatio-spectral EEG signal features are consistent with prior studies [29,30]. Consequently, idling and dorsifiexion epochs could be predicted from the underlying multi-channel EEG data with an accuracy as high as 97.6%, and all subjects achieved performances that were significantly above random chance.

The results achieved online demonstrate that BCI-FES-mediated foot dorsifiexion can be reliably controlled using a contralateral control paradigm in a small population of able-bodied individuals. In general, this study suggests that the integration of a noninvasive BCI with a lower-extremity FES system is feasible. In addition to achieving excellent performances, all subjects were able to assume immediate control of the interface, requiring only a single, short 20 min training session to develop a prediction model, and a single 3 min calibration session (see Methods section). It should be noted that the high performances achieved offline generally transferred into robust online BCI-FES operation, indicating that our cross-validation procedure selected the correct prediction model without overfitting. It should also be noted that since the prediction model was designed based on data largely free of any systematic artifacts, activities such as teeth clenching, grimacing, and other forms of "cheating" are likely to be ineffective in the online session, since the model was not designed to recognize them. Similarly, other electrophysiological artifacts of cortical origin (e.g. epileptic discharges) are unlikely to affect the system's online performance. Finally, the FES-elicited movements during online operation did not interfere with the control of the BCI system. For example, upon cessation of voluntary foot dorsifiexion, it is conceivable that EEG signals due to FES-elicited movements may be confused with those of voluntary movements, which may in turn confuse the classifier and cause the system to remain in the dorsifiexion state. This type of positive feedback, however, was not observed, perhaps because the EEG signals underlying these types of movements were sufficiently different and did not get misclassified. These differences may reflect spatial separation of cortical representations of FES-induced passive movements (likely localized to sensory cortex areas) and of voluntary movements (originating from more anterior brain motor areas).

### Future Directions

This BCI-FES dorsifiexion system may be used in a future seated therapeutic exercise that can facilitate neural repair in stroke, SCI, or TBI patients who are affected by foot-drop. By pairing activation of motor cortex associated with attempted, but impaired dorsifiexion, with electrical stimulation of foot dorsifiexion motor pools (via antidromic electrical stimulation of the deep peroneal nerve), it can be hypothesized that spared connections between the post injury motor cortex and the motor pools of foot dorsifiexion will be reinforced in a Hebbian manner. This hypothesized plasticity process associated with BCI-FES use may thereby translate into the improvement of unassisted dorsifiexion strength and gait function in this patient population. Since many patients with stroke, TBI, or SCI may have non-classical foot motor representation due to post-injury reorganization, the use of the data-driven method described in the current work to generate a "personalized" EEG prediction model for each subject will be of particular importance.

While the performance of the current system was tested in a contralaterally-controlled FES paradigm, its practical application in individuals with paralysis due to neurological injury will require utilization of an ipsilaterally-controlled FES paradigm, whereby attempted movement of the paralyzed limb acts as the control strategy for its own BCI-FES mediated movement. Additional studies will need to be undertaken in individuals with foot-drop due to central neurological injury in order to assess the utility of this paradigm.

The hypothesized applicability towards neuro-rehabilitation by utilizing an ipsilaterally-controlled FES paradigm to induce Hebbian neural recovery raises significant concerns about eliminating the latency between the onset of voluntary movement and BCI-FES mediated movement. The observed latency is partly caused by the averaging of posterior probability over 1.5 sec period (see Calibration section) during online operation. Reducing the averaging window may help decrease this latency, although perhaps at the expense of lowering the online performance (higher false positive and omission rates). Note that the observed latency is also consistent with a natural delay of maximal event-related desynchronization and synchronization of EEG sensorimotor rhythms [30,32], and it may be partially responsible for the delay in the BCI-FES system response. A potential solution to this problem may be to use our data-driven algorithm to search for relevant time domain EEG features, such as readiness potentials [33]. These slow negative potential shifts may be observed as early as 1 sec before the initiation of a self-paced motor behavior [33], and they can potentially be used for early classification of dorsifiexion and idling. Also, a combination of temporal and spectral features may be used in the future to eliminate the latency while ensuring high performance. However, further research is required, as changes in both the training paradigm and signal processing methodology will need to be implemented such that novel EEG features associated with movement intentions can be reliably detected.

Finally, the ideal BCI-FES system should mimic foot dorsifiexion in a 1:1 temporal fashion, in which a single foot dorsifiexion cycle (as opposed to current repetitive dorsifiexion) translates into a single BCI-FES mediated dorsifiexion cycle. To achieve this, changes in both the training paradigm and signal processing methodology will need to be implemented such that transient EEG changes associated with foot dorsifiexion and relaxation can be reliably detected and utilized to govern the BCI-FES system's state transitions. In summary, all of the above improvements and revisions would conceivably result in a more intuitive BCI neuroprosthesis and could lead to a seated therapeutic foot dorsifiexion exercise for individuals who suffer from foot-drop due to a central nervous system injury.

## Conclusions

The present study demonstrates that the integration of a noninvasive EEG-based BCI system with a noninvasive FES system for the lower extremities is feasible. The integrated BCI-FES system shows that EEG signals can be used to enable direct brain control of foot dorsifiexion via FES. This further suggests that it may be feasible to utilize BCI-FES systems to restore lost motor function of the lower extremities in patients with neurological injury. While the performance of the current system was tested in a contralaterally-controlled FES paradigm, its practical application in individuals with paralysis due to neurological injury will require the utilization of an ipsilaterally-controlled FES paradigm, whereby attempted movement of the paralyzed limb acts as the control strategy for its own BCI-FES mediated movement. Despite the presence of neurological injury (e.g. stroke, SCI, or TBI), and regardless of its extent and location, EEG spatio-spectral signal features discriminating attempted foot dorsifiexion and resting states are still expected to be present [34,35] and exploitable for BCI control by using a data-driven method for generating "personalized" prediction models. If the elimination of delay and the implementation of a 1:1 attempted ipsilateral-BCI-FES dorsifiexion paradigm are successful, BCI-FES systems will likely prove useful in the neuro-rehabilitation of individuals with lower extremity paralysis due to neurological injury.

## Competing interests

The authors declare that they have no competing interests.

## Authors' contributions

AHD conceived the study and the design of the BCI-FES system, oversaw the experiments, and co-wrote the article. PTW and CEK carried out the experiments, collected and analyzed data, and proofread the article. PTW also programmed the brain-computer interface software. AA designed and implemented the hardware integration between the BCI computer and the FES system, and programmed the microcontroller. ZN designed the signal processing, pattern recognition, and classification algorithms, analyzed the online performance, and co-wrote the article. All authors read and approved the final manuscript.

## Endnotes

^1^The conditional probability of event *A *given event *B*, denoted by *P*(*A|B*), is the probability of the event *A *given that the event *B *has happened. The posterior probability of idling class, denoted by $P\left(I|f^{⋆}\right)$, is the conditional probability of idling class given that the value *f*^⋆ ^of the feature *f *was observed. The posterior probability of dorsifiexion class, $P\left(D|f^{⋆}\right)$, is defined similarly.
